# Supplementary figures and images for: Evidence for a novel, effective approach to targeting carcinoma catabolism exploiting the first-in-class, anti-cancer mitochondrial drug, CPI-613
Source: PLoS One. 2022 Jun 8;17(6):e0269620. doi: 10.1371/journal.pone.0269620 (PMC9176802; doi:10.1371/journal.pone.0269620)

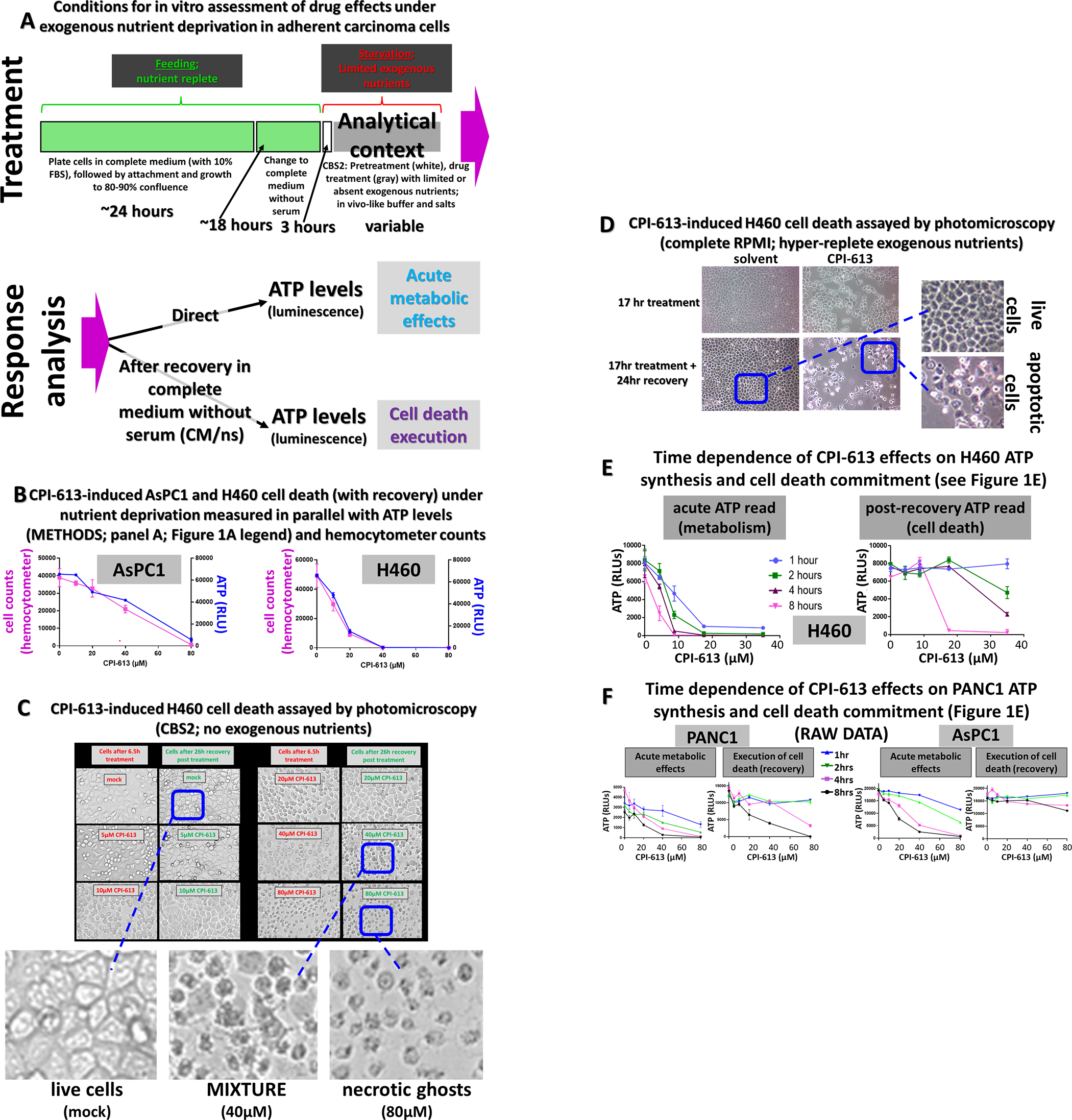

Supplement: S1 Fig — A. Analytic procedure and validation of cell death assay in adherent carcinoma lines, part 1. A: Flow diagram for analysis of drug effects on acute ATP levels (metabolism) and commitment to/execution of drug-induced cell death in the absence (or controlled presence) of exogenous nutrients (see Materials and Methods for additional details). The serum-free pretreatment steps diagrammed produce cell cycle arrest, but leave cells metabolically active. In the absence of exogenous nutrients and accelerated, drug-induced starvation (text), these metabolically active carcinoma cells ultimately exhaust endogenous nutrient stores, undergoing passive starvation and cell death indistinguishable from the drug-induced process. The time to this passive starvation varies between cell lines, ranging from ~15 hours for the highly sensitive PC3 cell line to ~2 days for H460 and PANC1; and ~5 days for AsPC1. It remains to be determined how this procedure might require modification for application to leukemia or lymphoma cells. The main effects analyzed in this paper are highly reproducible, in spite of modest experiment-to-experiment quantitative variation (see, for example, Fig 1E). This variation likely results from small differences in cell density (and, thus, nutrient availability) during the complete medium pre-feeding step. B-C. Analytic procedure and validation of cell death assay in adherent carcinoma lines; part 2. B. Cells were plated and grown as indicated in panel A and the legend to Fig 1A. Drug treatment time was 6.5hrs (H460) or 20hrs (AsPC1). Cells were photographed (H460; panel C), then incubated for an additional 25hrs in CM/ns and again photographed (panel C). One duplicate plate was then analyzed for survival by ATP level measurement and the other by hemocytometer cell counts (Supplementary Materials and Methods in S1 File). Note that these cells were treated in the absence of exogenous nutrients (CBS2; see Fig 1A). The elevated values of ATP RLUs in this experiment [file pone.0269620.s001.tif]

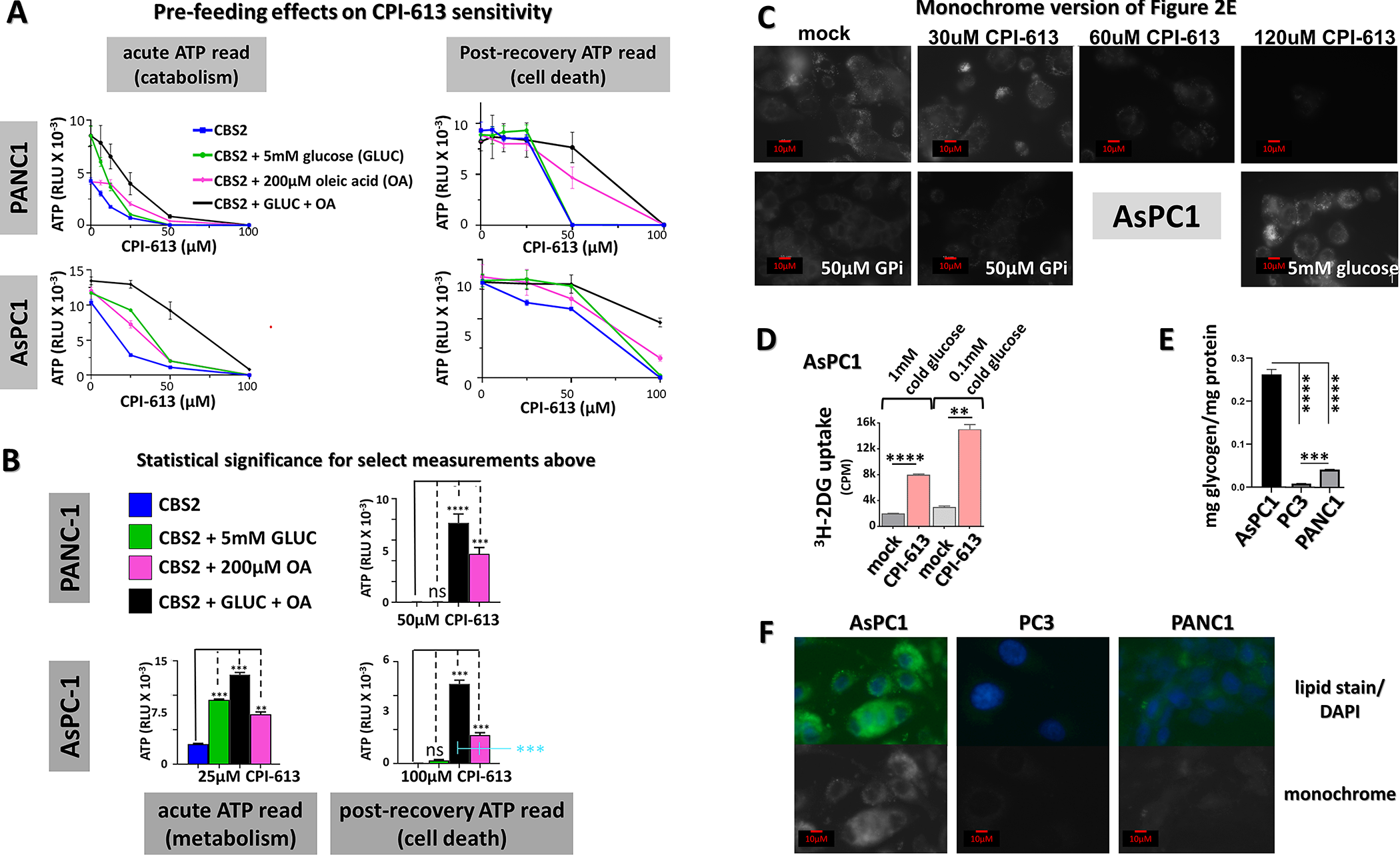

Supplement: S2 Fig — A-B Fig. Pre-feeding of cells designed to enhance endogenous nutrient stores can strongly affect CPI-613 response. A: Effects of pre-feeding PANC1 and AsPC1 cells with GLUC and/or OA on resistance to subsequent CPI-613-induced acute ATP loss and commitment to cell death in the absence of exogenous nutrients. If endogenous nutrient store depletion is necessary for CPI-613-induced reduction in ATP levels and carcinoma cell death commitment, enhancing such stores should increase carcinoma cell resistance to the drug. To test this prediction, we investigated the drug response of PANC1 and AsPC1 cells under identical treatment conditions after differential pre-feeding with glucose (GLUC) and/or oleic acid (OA). These actions are designed to enhance carbohydrate and/or lipid stores, respectively (Fig 2B–2D; main text). Again, all CPI-613 treatment conditions shown in this experiment are identical; only the immediate history of the cells’ prior exposure to exogenous glucose and/or oleic acid distinguishes the various samples. Pre-feeding has robust effects on both cell lines, providing significant protection from CPI-613 effects both on acute ATP levels and/or ensuing commitment to cell death. Moreover, the differences in the details of these responses in the two cell lines are consistent with AsPC1 having higher intrinsic levels of nutrient stores (before pre-feeding) and/or acquiring higher store levels during pre-feeding; in contrast, the behavior of the sensitive PANC1 line is consistent with its having and/or acquiring lower nutrient store levels. Specifically, cells were pre-fed for 5hrs with the indicated concentrations of glucose and/or oleic acid in CBS2 immediately before drugging in nutrient-free CBS2 (S1A Fig). Exogenous nutrient-containing medium was removed, cells were washed, and then treated with the indicated concentrations of CPI-613 for 10hrs in CBS2. At the end of this treatment, ATP levels were measured directly (left; predominantly acute metabolic eff [file pone.0269620.s002.tif]

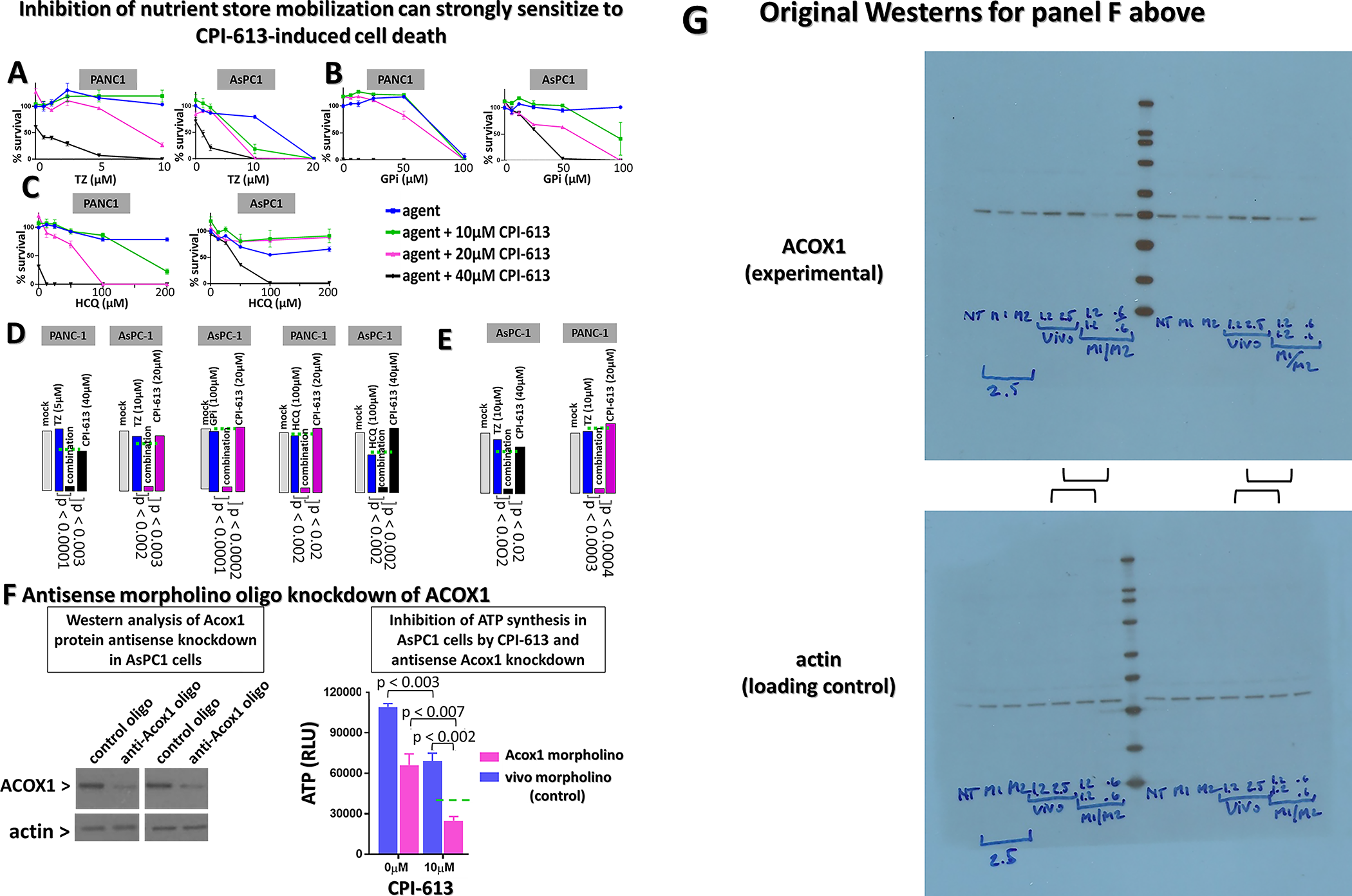

Supplement: S3 Fig — A-F Fig. Additional plots of nutrient store inhibitor enhancement of CPI-613 potency. A-C: Reference copies of data in Figs 3A and 5A in main text, as well as additional data showing PANC1 TZ response under conditions described in legend to Fig 3A. D-E: Statistical significance for data in bar graph interaction plots as described in legend to Fig 3A. Dashed green lines indicate the combination value predicted if the two indicated agent levels act independently of one another. F: ACOX1 protein levels were knocked down with antisense morpholino oligos at left (Supplementary Materials and Methods in S1 File). Control oligo was used at 2.5μM and the anti-ACOX1 oligos were a mixture of two independently targeting the ACOX1 message (each at 1.25μM). ATP levels were assayed after antisense treatment followed by exposure to indicated CPI-613 concentrations for 19hrs at right (Supplementary Materials and Methods in S1 File). [Higher RLU values observed in this experiment result from the use of reflective white plates, due to supply chain constraints. Absorbent black plates were used for all other experiments herein except S1B Fig] Note that the morpholino antisense procedure induces some CPI-613 sensitization independently of the effects of ACOX1 knockdown (compare to Fig 3A for example), most likely indicating metabolic stress induced by the antisense treatment procedure, itself, independently of ACOX1 targeting. Dashed green line indicates the combination value predicted if CPI-613 and the ACOX1 antisense oligo act independently of one another. G. Original Westerns from which the data in panel S3F Fig above were extracted. The wells used in the panel above are indicated by the brackets. (TIF) [file pone.0269620.s003.tif]

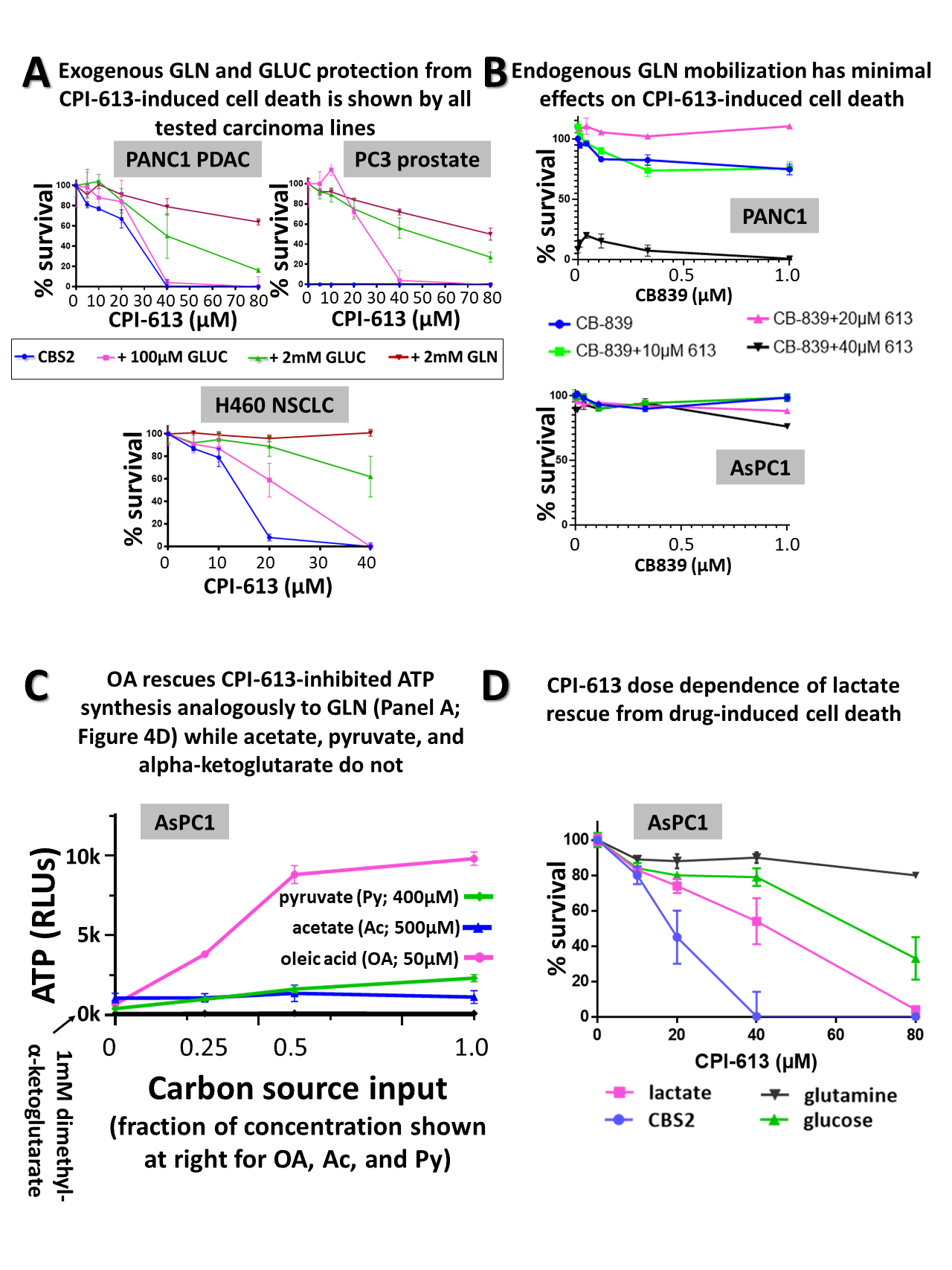

Supplement: S4 Fig — A. These experiments were carried out as described in Fig 1A, including 19-22hr post-treatment CM/ns recovery to assess cell death commitment. Initial drug treatment times were 15hrs for all cell lines. [Note that PC3 cells undergo passive starvation and death in the absence of exogenous carbon under these conditions.] B. CB839 inhibition of glutaminase GLN mobilization produces limited effects on CPI-613-induced cell death in the absence of exogenous nutrients, particularly in AsPC1. Cells were treated in CBS2 in the presence of CB839 for 15hrs, followed by 28hrs CM/ns recovery to assess cell death (see legend to Fig 1A). CB839 concentrations used span the range necessary to fully inhibit glutaminase activity in carcinoma cells (see Fig 4B, for example). C. OA rescues CPI-613-induced (60μM) ATP depletion in the presence of GPi (60μM) in a concentration dependent manner (6hr treatment). Other tested carbon sources not providing electrons directly to the ETC do not (acetate, dimethyl-alpha-ketoglutarate, and pyruvate). [Note that pyruvate oxidation is also blocked by CPI-613 effects on PDH.] The statistical significance (p value) for the differences between 400μM acetate and 50μM oleic acid is <0.0002 and for 400μM pyruvate and 50μM oleic acid is <0.0006. D. Unlike the glutamine GDH electron pair, the extra lactate electron pair (relative to pyruvate) cannot rescue from CPI-613-induced cell death at a robust drug dose (80μM; in the presence 50μM GPi; 16hrs treatment, 22hrs recovery, as in Fig 1A). At the lower 40μM CPI-613 dose lactate produces modest rescue. Both these results are as expected (text). Carbon sources were 1mM glucose, 2mM glutamine, and 2mM lactate. The statistical significance (p value) for the differences between lactate and glutamine and lactate and glucose at 80μM CPI-613 are <0.002 and <0.02, respectively. This significance value is <0.02 for the difference between lactate and CBS2 at 40μM CPI-613. (TIF) [file pone.0269620.s004.tif]

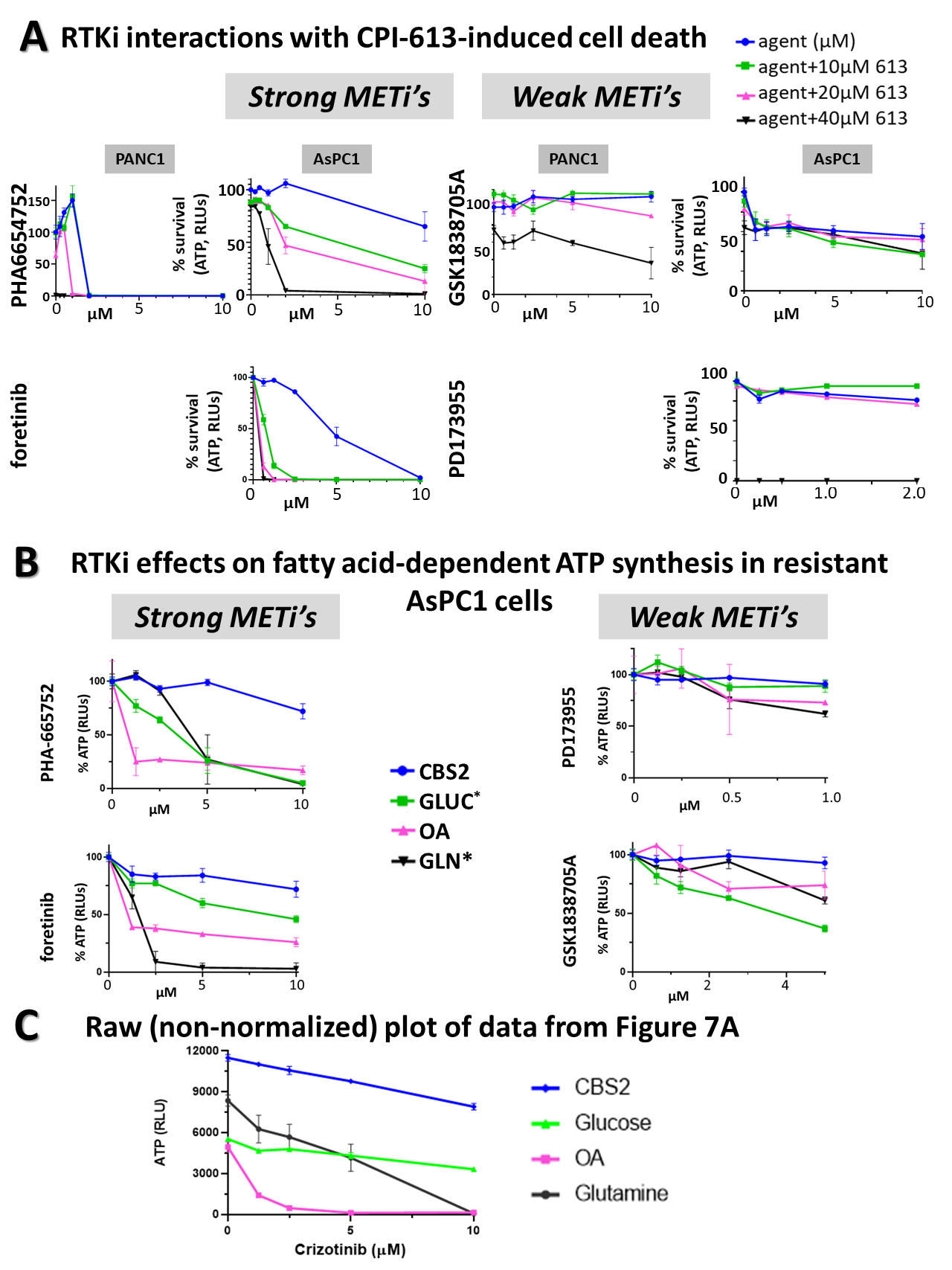

Supplement: S5 Fig — A: Tested high affinity MET inhibitors (left and Fig 7B) robustly sensitize to CPI-613-induced cell death (resistance dependent on endogenous nutrient stores). In contrast, the two tested low affinity MET inhibitors (right) show more limited sensitization (see Fig 7C for inhibitor affinities). These patterns of sensitization are also supported by the acute data in panel B. Assessment of cell death (treatment followed by CM/ns recovery; Fig 1A; Materials and Methods) induced by the indicated combinations of CPI-613 and RTKi’s during 15hrs of treatment followed by 20–24 hrs recovery (reference Fig 7B). Note that PHA6654752 induces a substantial, stable elevation of ATP levels at low doses in PANC1, presumably reflecting an RTK regulatory effect. B. High affinity MET inhibitors (left and Fig 7A) show robust inhibition of acute ATP synthesis driven by OA. In contrast, the two tested low affinity MET inhibitors (right) show little or no inhibition of OA-dependent ATP synthesis. Also see legend to Fig 7A and Figs 3D, 4B, and S4C Fig for additional technical details (50μM GPi; 60μM CPI-613; 50μM oleic acid; 1mM glucose; 1mM glutamine; treatment time 3 hours) and secondary controls. Each of the strong METi’s also has a distinct pattern of effects on ATP synthesis driven by glucose and glutamine (*). These distinctions presumably reflect other metabolic targets idiosyncratic to each of these regulatory agents. Dose range for each drug was chosen on the basis of its effects in assays in the published literature. For convenience of visualization, each data line is normalized to its own mock treated point, analogously to Fig 2E. C. Raw data from Fig 7A, plotted without normalization. (TIF) [file pone.0269620.s005.tif]
