# Supplementary material for: Evidence for a novel, effective approach to targeting carcinoma catabolism exploiting the first-in-class, anti-cancer mitochondrial drug, CPI-613
Source: PLoS One. 2022 Jun 8;17(6):e0269620. doi: 10.1371/journal.pone.0269620 (PMC9176802; doi:10.1371/journal.pone.0269620)
Supplement: S1 File — (DOCX) [file pone.0269620.s006.docx]

**Supporting Information**

**Supplementary Figures (at end of manuscript text)**

**Supplementary Materials and Methods**

**(See Supplementary References)**

**Acute ATP measurements and carcinoma cell survival measurements**

LOGIC (also see Materials and Methods and assay validation in S1A-D Fig): High throughput, plate reader assays for cell death commonly ultimately depend on metabolic activity of cells to identify viability, directly or indirectly. Thus, strong inhibition of metabolic activity by anti-metabolism agents like those explored in this work can confound cell death analysis by such assays. To overcome this limitation, we took advantage of the fact that the agents we use herein ultimately inhibit mitochondrial metabolism, but not glycolysis. We can, thus, use a recovery-based assay wherein cells are treated with anti-metabolic agents under conditions where mitochondrial metabolism is (or becomes) limiting on viability. After experimental treatment, drug(s) and media are removed and cells are allowed to recover in serum-free complete medium (CM/ns). Complete media provide high levels of glucose (11mM) in support of glycolytic ATP generation (independently of mitochondrial function; Results), allowing viable cells to recover ATP levels that are ultimately measured in the plate assay to score cell viability. In the absence of serum, viable cells do not replicate. Under these conditions, cells that are committed to execution of cell death by the experimental treatment score as dead and cells that have not committed to cell death recover ATP levels and score as viable (S1A-D Fig; also see 11).

Specifically, cells are exposed to the treatments indicated in the text and figure legends, followed by recovery (17-24 hours) in CM/ns. Survival is assessed by luminescence measurement of ATP levels after this recovery (CellTiter-GLO, Promega).

We have validated this assay extensively in multiple independent ways, including FACS analysis and video microscopy (11). We have also validated this assay by comparing hemocytometer cell counts and ATP cell survival measurements (see examples in S1B Fig). Moreover, we commonly spot check recovery/ATP assays of cell death by direct microscopy (examples of what these assays show are in S1C-D Fig). Also see Figs 2E,4B and S1E,S2A Fig for illustrations of the effectiveness of this approach in distinguishing metabolic inhibition from commitment to and execution of cell death.

Details of assay validation (S1B-C Fig): Cells were plated at 40,000 per well in duplicate black, clear bottom plates in complete medium supplemented with 10% FBS and grown for 20 hours. At this time, the plating medium was replaced with CM/ns (containing 11mM glucose) and incubated for 15hr. Cells were then incubated is CBS2 for 3 hours. CBS2 was replaced with CBS2 plus CPI-613 at concentrations indicated in figures (in triplicate wells). H460 cells and AsPC1 cells were incubated for 6 or 20 hours, respectively. H460 cells were photographed at the end of the six hour incubation and after recovery (S1C Fig). Images were collected with EVOS*fl* digital inverted microscope (AMG), 10X objective. All vehicle or CPI-613-containing medium was replaced with CM/ns and incubated for an additional 24 hours. At the end of recovery, one plate was used to measure ATP (CellTiter-GLO, Promega) and the duplicate plate was used to count cells (hemocytometer)(S1B Fig).

Details of cell counting (S1B Fig): Wells were processed in sets of three corresponding to each treatment condition. 40 µL of 0.25% trypsin-EDTA (Invitrogen) was added to the CM/ns and incubated for 12-15 minutes at 37^o^C. Cells were dislodged and mixed by pipetting up and down 4 times to obtain a uniform cell suspension. 10ul of the suspension was transferred to the trough of a hemocytometer, the cell suspension was drawn into the chamber by capillary action and cells were counted. Note that necrotic cell residues (S1C Fig) fragment extensively under these conditions and are not visible for hemocytometer counting.

**Steady state metabolite levels determination**

The steady state metabolomics analysis in Fig 1C used well established assays and procedures in collaboration with Metabolon, Inc. (Durham, NC, USA). This study was described in detail and other results from the study were reported from in 12.

In brief, samples were extracted and split into equal parts for analysis on the gas chromatography-mass spectrometry and liquid chromatography-tandem mass spectrometry platforms (Evans, et al., 2009). Proprietary software was used to match ions to an in-house library of standards for metabolite identification and for metabolite quantitation by peak area integration (DeHaven, et al., 2010). Extracts were prepared according to Metabolon’s standard methanol-based extraction protocol. Samples were analyzed on a Thermo-Finnigan Trace DSQ fast-scanning single-quadrupole mass spectrometer (Waltham, MA) using electron impact ionization.

Cell pellet and medium preparations: Cell preparation was as follows. 3.5x10^6^ BxPC3 PDAC cells were plated in 10cm plates in culture medium supplemented with 10% FBS and 100U/ml penicillin and 100ug/ml streptomycin. Cells were allowed to grow for two days reaching 90-95% confluence. At this time, the medium was replaced with fresh medium containing solvent (0.075% dimethylformamide) or 240μM CPI-613 in solvent and treated for times indicated in Fig 1C. At the end of treatment, plates were placed on ice, medium was removed and retained for analysis. Cells were washed with 3mls of ice-cold serum-free complete medium. 3mls of fresh ice-cold medium was added to the plates, cells were scraped and transferred into 15 ml semiconical tubes and centrifuged at 180g for 3 minutes. Supernatant was removed, samples were spun for an additional 30 seconds and all residual medium was removed. Cell pellets were flash frozen in liquid nitrogen and stored at -80°C until shipment to Metabolon for biochemical profiling.

Global untargeted biochemical profiling: The LC/MS portion of the platform was based on a Waters ACQUITY UPLC and a Thermo-Finnigan LTQ mass spectrometer, which consisted of an electrospray ionization (ESI) source and linear ion-trap (LIT) mass analyzer. The sample media or cell pellet extract was split into two aliquots, dried, then reconstituted in acidic or basic LC-compatible solvents, each of which contained 11 injection standards at fixed concentrations. One aliquot was analyzed using acidic positive ion optimized conditions and the other using basic negative ion optimized conditions in two independent injections using separate dedicated columns. Extracts reconstituted in acidic conditions were gradient eluted using water and methanol both containing 0.1% Formic acid, while the basic extracts, which also used water/methanol, contained 6.5mM ammonium bicarbonate. The MS analysis alternated between MS and data-dependent MS scans using dynamic exclusion. Chromatographic separation was performed, followed by full-scan mass spectroscopy, to record and quantify all detectable ions presented in the samples. Metabolites with known chemical structure were identified by matching the ions’ chromatographic retention index and mass spectral fragmentation signatures with reference library entries created from authentic standard metabolites under the identical analytical procedure as the experimental samples.

An aliquot of each experimental media sample was taken then pooled for the creation of “Client Matrix” (CMTRX) samples. These CMTRX samples were injected throughout the platform run and served as technical replicates, allowing variability in the quantitation of all consistently detected endogenous biochemicals to be determined and overall process variability and platform performance to be monitored.

Statistical analysis: Data normalization was performed to correct variation resulting from instrument inter-day tuning differences. Each compound was corrected in run-day blocks by registering the medians to equal one (1.00) and normalizing each data point proportionately. Missing values (if any) were assumed to be below the level of detection for that biochemical with the instrumentation used and were imputed with the observed minimum for that particular biochemical. After normalization and imputation, the data were log-transformed. *t* tests were then performed to compare the treatment conditions as well as the time points across treatments. Multiple comparisons were accounted for with the false discovery (FDR) rate method, and each FDR was estimated by q-values (12).

**2-deoxyglucose uptake (Fig 1D)**

Seeding, drug treatment, and 2DG uptake were performed in complete media supplemented with 10% FBS for BxPC3, H460, PANC1, and AsPC1. All treatments were done in triplicate (biological replicates). BxPC3 cells were seeded at 500,000 cells per 35-mm dish; PANC1, AsPC1, and H460 were plated in 6 well plates at 300,000 cells per well for PANC1 and at 500,000 cells per well for AsPC1 and H460. Following a 48hr incubation, seeding medium was replaced with fresh medium and incubated for 2 hrs. At this time, solvent (control) or CPI-613 (240µM) were added to the medium and incubated for 2 hours. One hour after the addition of solvent or CPI-613, 1µCi of 1,2-^3^H-2-deoxyglucose (Moravek Biochemicals) was added to the medium. Thus, all samples received a 1hr labeling pulse. To minimize exposure of cells to extreme surface tension, all additions of solvent, CPI-613, and 2-deoxyglucose were done by removing ½ of medium and replacing with equivalent media volume containing appropriate concentration of agents.

At the end of the 2 hour drug treatment, plates were placed on ice, medium removed and plates were washed three times with ice cold medium without FBS. 300µl of 0.5% triton in PBS was added to each plate/well and plates were incubated at room temperature for 10 minutes. Lysates were transferred into scintillation vials containing 3mls of scintillation cocktail and counted.

### **Thioridazine and etomoxir inhibition of CO_2_ release (Fig 3C)**

AsPC1 cells were seeded in 12 well plates at 500,000 cells per well in complete medium supplemented with 10 % FBS and incubated for 48 hours. At this time, the seeding medium was replaced with CM/ns and incubated 20hrs. The CM/ns was replaced with 700µL CBS2 supplemented with 40μM oleic acid (OA) and 50μM CP91149 (glycogen phosphorylase inhibitor, GPi) and cells were incubated for 3 hours. After three-hour incubation, thioridazine or etomoxir were added at the concentrations indicated in the figure and samples were incubated for an additional 1 hour. 0.1μCi of 1-^14^C labeled oleic acid (Moravek Biochemicals) was then added to each well and incubated for an additional 30 minutes, resulting in a 30-minute labeling pulse. The samples were terminated by addition of 1M perchloric acid to a final concentration of 150mM. In addition to killing the cells and terminating biochemical reactions, the acidification of the medium results in the release of CO_2_ from sodium bicarbonate in the medium. The released CO_2_ is captured in phenyl ethyl amine saturated filters placed on top of each well. Collection of released CO_2_ is done for ~20hrs, at which time the filters are counted.

### **Morpholino antisense oligo knockdown of ACOX1 (S3F Fig)**

Western analysis of ACOX1 knockdown: 50,000 AsPC-1 cells/well were plated in 24 well plates in complete medium in peripheral wells. 24hrs post plating, seeding medium was replaced with 0.5ml CM/ns + 2.5µM total of ACOX1 Morpholino (MO) 1:1 mixture or Vivo-Control and incubated for 6hrs. Morpholino treatment was terminated by replacing morpholino solutions with complete medium and incubated for an additional 48 hrs.

Cells were lysed with RIPA buffer. Acox1 levels in lysates were analyzed via Western blot antibodies against ACOX1 (AbCam ab184032) and beta actin (Sigma A5060) (S3F Fig).

Morpholino oligos were obtained from Gene Tools, LLC, Philomath, OR.

Morpholino sequences: MO1 sequence: 5’-CTGGCAGCGAAGTAACGACCGACC,

MO2 sequence: 5’-GCAGTGACAATCTAAATCCGCAGCT,

Vivo-Control sequence: 5’-CCTCTTACCTCAGTTACAATTTATA

ATP assay:

10,000 AsPC-1 cells/well were plated in 96 well plates in complete medium.

Morpholino treatment: 24h post plating, seeding medium was replaced with 50µl CM/ns + 2.5uM total of ACOX1 Morpholino (MO) mixture or Vivo-Control and incubated for 8 hrs.

Morpholino treatment was terminated by replacing morpholino solutions with complete medium.

Assessment of ATP reduction by ACOX1 knockdown and/or CPI-613 treatment: 2 days post morpholino treatment, complete medium was replaced with CM/ns and incubated for 18hrs. CM/ns was then replaced with CBS2+50µM CP91,149 [glycogen phosphorylase inhibitor, (GPi)] and incubated for 3hrs. CBS2/GPi was replaced with fresh CBS2/GPi or CBS2/GPi + CPI-613 and incubated for 19hrs. ATP was assayed with CellTiterGLO (Promega).

**Reagents and chemicals**

Nutrients:

|  |  |  |
| --- | --- | --- |
| **Chemical** | **Vendor** | **Catolog #** |
| D-(+)-Glucose Solution (45%) | Sigma | G8769 |
| L-Glutamine (200mM) | ThermoFisher Scientific-Gibco | 25030-081 |
| Oleic Acid -Water Soluble | Sigma | O1257-10MG |
| Dimethyl-alpha-ketoglutarate | Sigma | 349631-5G |
| Acetate (sodium acetate trihydrate) | Sigma | S-8625 |
| Sodium Pyruvate (100mM) | ThermoFisher Scientific-Gibco | 11360-070 |
| N-Acetyl-L-cysteine | Sigma | A8199-10G |
|  |  |  |

Chemicals/drugs:

Pharmaceutical grade CPI-613 was provided for the studies herein by Rafael Pharmaceuticals.

|  |  |  |
| --- | --- | --- |
| **Chemical** | **Vendor** | **Catolog #** |
| Crizotinib | ApexBio | A3020 |
| PHA-665752 | ApexBio | A2307 |
| Thioridazine | Cayman Chem | 14400 |
| CP-91149 | Selleck eChem | S2717 |
| Hydroxychloroquine | TCI | H1306 |
| PD 0325901 | Cayman Chem | 13034 |
| Hexachlorophene | Cayman Chem | 23948 |
| BAM15 | Cayman Chem | 17811 |
| (+)-Etomoxir | Cayman Chem | 11969 |
| CB-839 | Cayman Chem | 22038 |
| Phenformin | Cayman Chem | 14997 |
| Foretinib | ApexBio | A2974 |
| GSK1838705A | Cayman Chem | 24904 |
| Rotenone | Sigma | R8875-1G |
|  |  |  |

**Supplementary References**

**Introduction**

Alistar A, Morris B B, Desnoyer R, Klepin HD, Hosseinzadeh K, Clark C , et al. Safety and tolerability of the first-in-class agent CPI-613 in combination with modified FOLFIRINOX in patients with metastatic pancreatic cancer: a single-centre, open-label, dose-escalation, phase 1 trial. *Lancet Oncology* 2017;18:770-778. doi:10 1016/s1470-2045(17)30314-5

Galluzzi L , Kepp O, Vander Heiden MG, Kroemer G. Metabolic targets for cancer therapy. *Nature Rev Drug Disc* **2013**;12:829-846. doi 10.1038/nrd4145

Garcia-Canaveras JC, Lahoz A. Tumor microenvironment-derived metabolites: A guide to find new metabolic therapeutic targets and biomarkers. *Cancers* **2021**;13:3230. doi:10.3390/cancers13133230

Guo JY, Teng X, Laddha SV, Ma SR, Van Nostrand SC, Yang Y. Autophagy provides metabolic substrates to maintain energy charge and nucleotide pools in Ras-driven lung cancer cells. *Genes & Dev* **2016**;30:1704-1717. doi:10.1101/gad.283416.116

Hanahan D, Weinberg RA. Hallmarks of cancer: the next generation. *Cell* **2011**;144:646-674. doi:10 1016/j cell 2011 02 013

Hay N. Reprogramming glucose metabolism in cancer: can it be exploited for cancer therapy? *Nat Rev Can* **2016**;16:635-649. doi:10.1038/nrc.2016.77

Hensley CT, Faubert B, Yuan Q, Lev-Cohain N, Jin E, Kim J, et al. Metabolic heterogeneity in human lung tumors. Cell **2016**;164:681-694. doi:10.1016/j.cell.2015.12.034

Kamphorst J J, Nofal M, Commisso C, Hackett SR, Lu WY, Grabocka E, et al. Human pancreatic cancer tumors are nutrient poor and tumor cells actively scavenge extracellular protein. Can Res 2015;75:544-553. doi:10.1158/0008-5472.Can-14-2211.

Koukourakis MI, Giatromanolaki A, Harris AL, Sivridis E. Comparison of metabolic pathways between cancer cells and stromal cells in colorectal carcinomas: a metabolic survival role for tumor-associated stroma. *Can Res* **2006**;66:632-637. doi:10.1158/0008-5472.can-05-3260

Krall AS, Mullen PJ, Surjono F, Momcilovi M, Schmid EW, Halbrook CJ, et al. Asparagine couples mitochondrial respiration to ATF4 activity and tumor growth. *Cell Metab* **2021**;33:1013-1026. doi:10.1016/j.cmet.2021.02.001

Martinez-Outschoorn UE, Peiris-Pages M, Pestell RG, Sotgia F, Lisanti MP. Cancer metabolism: a therapeutic perspective. *Nat Rev Clin Onc* **2017**;14:11-31. doi:10.1038/nrclinonc.2016.60

Mayers JR, Vander Heiden MG. Famine versus feast: understanding the metabolism of tumors in vivo. *Trends Bioch Sci* **2015**;40:130-140. doi:10.1016/j.tibs.2015.01.004

McFate T, Mohyeldin A, Lu H, Thakar J, Henriques J, Halim ND, et al. Pyruvate dehydrogenase complex activity controls metabolic and malignant phenotype in cancer cells. *J Biol Chem* **2008**;283:22700-22708. doi:10.1074/jbc.M801765200

McLain AL, Szweda PA, Szweda LI. α-Ketoglutarate dehydrogenase: A mitochondrial redox sensor. *Free Rad Res* **2011**;45:29-36. doi:10.3109/10715762.2010.534163

Milkovic L, Tomljanovic M, Gasparovic AC, Kujundzic RN, Simunic D, Konjevoda P, et al. Nutritional stress in head and neck cancer originating cell lines: the sensitivity of the NRF2-NQO1 axis. *Cells* **2019**;8:1001. doi:10.3390/cells8091001

Papandreou I, Cairns RA, Fontana L, Lim AL, Denko NC. HIF-1 mediates adaptation to hypoxia by actively downregulating mitochondrial oxygen consumption. *Cell Metab* **2006**;3:187-197. doi:10.1016/j.cmet.2006.01.012

Pardee TS, Anderson RG, Pladna KM, Isom S, Ghiraldeli LP, Miller LD, Powell BL. A Phase I study of CPI-613 in combination with high-dose cytarabine and mitoxantrone for relapsed or refractory acute myeloid leukemia. *Clin Can Res* **2018**;2:2060-2073. doi 10.1158/1078-0432.CCR-17-2282

Sai KKS, Zachar Z, Bingham PM, Mintz A. Metabolic PET imaging in oncology. *Am J Roentgenology* **2017**;209:270-276. doi:10.2214/ajr.17.18112

Timm KN, Kennedy BWC, Brindle KM. Imaging tumor metabolism to assess disease progression and treatment response. *Clin Can Res* **2016**;22:5196-5203. doi:10.1158/1078-0432.Ccr-16-0159

Vander Linden C, Corbet, C. Reconciling environment-mediated metabolic heterogeneity with the oncogene-driven cancer paradigm in precision oncology. *Sem Cell Dev Biol* **2020**;98:202-210. doi:10.1016/j.semcdb.2019.05.016

Vatrinet R, Leone G, De Luise M, Girolimetti G, Vidone M, Gasparre G, Porcelli AM. The α-ketoglutarate dehydrogenase complex in cancer metabolic plasticity. *Can & Metab* **2017**;5:3. doi:10.1186/s40170-017-0165-0

Viale A, Pettazzoni P, Lyssiotis CA, Ying HQ, Sanchez N, Marchesini M, et al. Oncogene ablation-resistant pancreatic cancer cells depend on mitochondrial function. *Nature* **2014**;514:628-632. doi:10.1038/nature13611

Wu CA, Chao Y, Shiah SG, Lin WW. Nutrient deprivation induces the Warburg effect through ROS/AMPK-dependent activation of pyruvate dehydrogenase kinase. *Bioch Biophys Acta-Mol Cell Res* **2013**;1833:1147-1156. doi:10.1016/j.bbamcr.2013.01.025

Yang SH, Wang XX, Contino G, Liesa M, Sahin E, Ying HQ et al. Pancreatic cancers require autophagy for tumor growth. *Genes & Dev* **2011**;25:717-729. doi 10.1101/gad.2016111

Ying HQ, Dey P, Yao WT, Kimmelman AC, Draetta GF, Maitra A, DePinho RA. Genetics and biology of pancreatic ductal adenocarcinoma. *Genes & Dev* **2016**;30:355-385. doi:10.1101/gad.275776.115

Ying HQ, Kimmelman AC, Lyssiotis CA, Hua SJ, Chu GC, Fletcher-Sananikone E, DePinho RA. Oncogenic KRAS maintains pancreatic tumors through regulation of anabolic glucose metabolism. *Cell* **2012**;149:656-670. doi 10.1016/j.cell.2012.01.058

Zhang M, Liu TT, Sun H, Weng WW, Zhang QY, Liu CC, et al. Pim1 supports human colorectal cancer growth during glucose deprivation by enhancing the Warburg effect. *Can Sci* **2018**;109:1468-1479. doi:10.1111/cas.13562

Zhang S, Hulver MW, McMillan RP, Cline MA, Gilbert ER. The pivotal role of pyruvate dehydrogenase kinases in metabolic flexibility. *Nutr & Metab* **2014**;11;10. doi:10.1186/1743-7075-11-10

**Results**

**FIG 1**

Davidson SM, Papagiannakopoulos T, Olenchock BA, Heyman JE, Keibler MA, Luengo A, et al. Environment impacts the metabolic dependencies of ras-driven non-small cell lung cancer. *Cell Metabolism* **2016**;23:517-528. doi:10.1016/j.cmet.2016.01.007

Flavahan WA, Wu QL, Hitomi M, Rahim N, Kim Y, Sloan AE, et al. Brain tumor initiating cells adapt to restricted nutrition through preferential glucose uptake. *Nat Neurosci* **2013**;16:1373-1376. doi:10.1038/nn.3510

Vaupel P, Hockel M. Blood supply, oxygenation status and metabolic micromilieu of breast cancers: Characterization and therapeutic relevance (Review). *Int J Onc* **2000**;17:869-879.

doi 10.3892/ijo.17.5.869.

**FIG 2**

Al-Bari MAA. Chloroquine analogues in drug discovery: new directions of uses, mechanisms of actions and toxic manifestations from malaria to multifarious diseases. *J Antimicro Chemo* **2015**;70:1608-1621. doi:10.1093/jac/dkv018

Boone BA, Bahary N, Zureikat AH, Moser AJ, Normolle DP, Wu WC, et al. Safety and biologic response of pre-operative autophagy inhibition in combination with gemcitabine in patients with pancreatic adenocarcinoma. *Ann Surg Onc* **2015**;22:4402-4410. doi:10.1245/s10434-015-4566-4

Gross MI, Demo SD, Dennison JB, Chen L, Chernov-Rogan T, Goyal B, et al. Antitumor activity of the glutaminase inhibitor CB-839 in triple-negative breast cancer. *Mol Can Ther* **2014**;13:890-901. doi:10.1158/1535-7163.Mct-13-0870

O'Connor RS, Guo LL, Ghassemi S, Snyder NW, Worth AJ, Weng L, et al. The CPT1a inhibitor, etomoxir induces severe oxidative stress at commonly used concentrations. *Sci Rep* **2018**;8:6289. doi:10.1038/s41598-018-24676-6

Raud B, Roy DG, Divakaruni AS, Tarasenko TN, Franke R, Ma EH, et al. Etomoxir actions on regulatory and memory T cells are independent of CPT1a-mediated fatty acid oxidation. *Cell Metab* **2018**;28:504-515. doi:10.1016/j.cmet.2018.06.002

Van den Branden C, Roels F. Thioridazine - a selective inhibitor of peroxisomal beta-oxidation in vivo. *FEBS Let* **1985**;187:331-333. doi:10.1016/0014-5793(85)81270-9

**FIGURES 3-7**

Aslostovar L, Boyd AL, Almakadi M, Collins TJ, Leong DP, Tirona RG, et al. A phase 1 trial evaluating thioridazine in combination with cytarabine in patients with acute myeloid leukemia. *Blood Adv* **2018**;2:1935-1945. doi:10.1182/bloodadvances.2018015677

Condello M, Pellegrini E, Caraglia M, Meschini S. Targeting autophagy to overcome human diseases. *Int J Mol Sci* **2019**;20:725. doi:10.3390/ijms20030725

Huyghe S, Mannaerts GP, Baes M, Van Veldhoven PP. Peroxisomal multifunctional protein-2: The enzyme, the patients and the knockout mouse model. *Bioch Biophys Acta-Mol Cell Biol Lip* **2006**;1761:973-994. doi:10.1016/j.bbalip.2006.04.006

Wanders RJA, Waterham HR, Ferdinandusse S. Metabolic interplay between peroxisomes and other subcellular organelles including mitochondria and the endoplasmic reticulum. *Front Cell Dev Biol* **2016**;3:83. doi:10.3389/fcell.2015.00083

**Discussion**

DeBerardinis RJ, Lum JJ, Thompson CB. Phosphatidylinositol 3-kinase-dependent modulation of carnitine palmitoyltransferase 1A expression regulates lipid metabolism during hematopoietic cell growth. *J Biol Chem* **2006**;281:37372-37380. doi:10.1074/jbc.M608372200

Bergers G, Fendt SM. The metabolism of cancer cells during metastasis. *Nat Rev Can* **2021**;21:162-180. doi:10.1038/s41568-020-00320-2

Duman C, Yaqubi K, Hoffmann A, Acikgoz AA, Korshunov A, Bendszus M, et al. Acyl-CoA-binding protein drives glioblastoma tumorigenesis by sustaining fatty acid oxidation. *Cell Metab* **2019**;30:274-289. doi:10.1016/j.cmet.2019.04.004

Elia I, Doglioni G, Fendt SM. Metabolic hallmarks of metastasis formation. *Trends Cell Biol* **2018**;28:673-684. doi:10.1016/j.tcb.2018.04.002

Harper ME, Antoniou A, Villalobos-Menuey E, Russo A, Trauger R, Vendemelio M, et al. Characterization of a novel metabolic strategy used by drug-resistant tumor cells. *FASEB J* **2002**;16:1550-1557. doi:10.1096/fj.02-0541com

Houten SM, Wanders RJA, Ranea-Robles P. Metabolic interactions between peroxisomes and mitochondria with a special focus on acylcarnitine metabolism. *Bioch Biophy Acta-Mol Basis Dis* **2020**;1866:165720. doi:10.1016/j.bbadis.2020.165720

Huang D, Li TT, Li XH, Zhang L, Sun LC, He XP, et al. HIF-1-mediated suppression of Acyl-CoA dehydrogenases and fatty acid oxidation is critical for cancer progression. *Cell Rep* **2014**;8:1930-1942. doi:10.1016/j.celrep.2014.08.028

Ma YB, Temkin SM, Hawkridge AM, Guo CQ, Wang W, Wang XY, Fan XJ. Fatty acid oxidation: An emerging facet of metabolic transformation in cancer. *Can Let 2018*;435:92-100. doi:10.1016/j.canlet.2018.08.006

Nagarajan SR, Butler LM, Hoy AJ. The diversity and breadth of cancer cell fatty acid metabolism. *Can & Metab* **2021**;9:2. doi:10.1186/s40170-020-00237-2

Padanad MS, Konstantinidou G, Venkateswaran N, Melegari M, Rindhe S, Mitsche M, et al. Fatty acid oxidation mediated by Acyl-CoA synthetase long chain 3 is required for mutant KRAS lung tumorigenesis. *Cell Rep* **2016**;16:1614-1628. doi:10.1016/j.celrep.2016.07.009

Sirois I, Aguilar-Mahecha A, Lafleur J, Fowler E, Vu V, Scriver M, et al. A unique morphological phenotype in chemoresistant triple-negative breast cancer reveals metabolic reprogramming and PLIN4 expression as a molecular vulnerability. *Mol Can Res* **2019**;17:2492-2507. doi 10.1158/1541-7786.MCR-19-0264

Viale A, Pettazzoni P, Lyssioti CA, Ying HQ, Sanchez N, Marchesini M, et al. Oncogene ablation-resistant pancreatic cancer cells depend on mitochondrial function. *Nature* **2014**;514:628-632. doi:10.1038/nature13611

Zaugg K, Yao Y, Reilly PT, Kannan K, Kiarash R, Mason J, et al. (2011). Carnitine palmitoyltransferase 1C promotes cell survival and tumor growth under conditions of metabolic stress. *Genes & Dev* **2011**;25:1041-1051. doi:10.1101/gad.1987211

**Supplementary Materials and Methods**

DeHaven CD, Evans AM, Dai H, Lawton KA. Organization of GC/MS and LC/MS metabolomics data into chemical libraries. *J Cheminform* **2010**;2:9. doi: 10.1186/1758-2946-2-9

Evans AM, DeHaven CD, Barrett T, Mitchell M, Milgram E: Integrated, nontargeted ultrahigh performance liquid chromatography/electrospray ionization tandem mass spectrometry platform for the identification and relative quantification of the small-molecule complement of biological systems. *Anal Chem* **200**;81:6656–6667. doi 10.1021/ac901536h
