# Supplementary material for: Evidence for a novel, effective approach to targeting carcinoma catabolism exploiting the first-in-class, anti-cancer mitochondrial drug, CPI-613
Source: PLoS One. 2022 Jun 8;17(6):e0269620. doi: 10.1371/journal.pone.0269620 (PMC9176802; doi:10.1371/journal.pone.0269620)
Supplement: Attachment — Submitted filename: anti-cancer mitochondrial drug, CPI-613.pdf [file pone.0269620.s007.pdf]

The authors Bingham and colleagues have studied CPI-613 as an anti-cancer mitochondrial drug in the manuscript titled *"Evidence for a novel, effective approach to targeting carcinoma catabolism exploiting the first-in-class, anti-cancer mitochondrial drug, CPI-613"*. The authors have investigated an exclusive targeting approach for altered metabolism of tumor cells and have shown interesting findings. The manuscript can be accepted without any revision.

The research findings of the study are promising and of high clinical relevance. The results display effectiveness in overcoming the limitations associated with targeting metabolic machinery including single-agent resistance by using multi-target approach with tumor-preferential inhibition of the mitochondrial tricarboxylic acid (TCA) cycle by CPI-613 (devimistat), a first-in-class drug. This translational approach can be investigated further for the advancement of current therapeutics.

The study offers strong evidence to support strategies for superior clinical targeting of carcinoma catabolism. However, authors show that cell line-specific, lipid stores in cancer cells may lead to CPI-613 resistance. Interestingly, using FDA approved agents, together with CP1-613 shows promising results in targeting resistant cells and may be highly beneficial in clinical setting in the future.

Targeting the fatty acid flow by clinically practical agents is possible and using such agents significantly sensitized a fully CPI-613-resistant carcinoma xenograft in vivo. The study also offers great advantage in understanding of the mechanistic pathways associated with resistance. However, heterogeneous effect of CPI-613 on different cells makes it more interesting as a candidate.
