# Supplementary material for: Evidence for a novel, effective approach to targeting carcinoma catabolism exploiting the first-in-class, anti-cancer mitochondrial drug, CPI-613
Source: PLoS One. 2022 Jun 8;17(6):e0269620. doi: 10.1371/journal.pone.0269620 (PMC9176802; doi:10.1371/journal.pone.0269620)
Supplement: Attachment — Submitted filename: Guardado Rivas, et al - RESPONSE TO REVIEWERS.docx [file pone.0269620.s008.docx]

Before addressing specific comments by the reviewers we note that we detected and corrected a quantitative clerical error in one treatment duration in the legend to Fig 4B. We emphasize that this correction has no impact on the interpretation of the results in this figure or any conclusions in the larger manuscript.

**REVIEWER 1:**

Reviewer 1 supported publication of our original manuscript without revision.

**REVIEWER 2:**

Reviewer 2 asks that we add data testing the generality of the glycogen and lipid store correlations with drug sensitivity characterized in PDAC cells lines. We have taken advantage of the exceptional sensitivity of the PC3 prostate cancer cell line to do this (see new figures S2E,F Fig; text lines 251-256). We find the predicted very low levels of glycogen and lipid stores in this very sensitive line. Collectively, our results robustly support the generality of the correlation between the levels of endogenous nutrient stores and CPI-613 sensitivity in carcinoma cells.

As requested by reviewer 2, we examined CPI-613 effects on glucose uptake under the nutrient depleted CBS2 conditions and correspondingly lower drug doses used in many of the experiments herein (see new figure S2D Fig; also see text lines 171-173). We find robust drug stimulation of glucose uptake under these conditions, analogous to those observed at higher drug doses in nutrient replete complete media (Fig 1C,D).

Reviewer 2 asked for further clarification of the significance of the three panels making up Figure 1E. We have edited the 1E legend to make this clearer (text lines 149-155).

Reviewer 2 asks about normal cell tolerance of CPI-613/TZ co-treatment. We enhanced our discussion of tumor selectivity of CPI-613 in lines 59-63 and of TZ in lines 491-493. We believe that the most reliable assessment of this issue comes from in vivo systems; thus, we have added additional comments on the current state of our knowledge about normal tissue responses to CPI-613/TZ combinations in the legend to Fig 6 (lines 530-533).

Reviewer 2 asks for clarification of several issues covered in Fig 6. We have extensively revised the presentation of these results. We improved the plotting of data in the lower left panel Fig 6B (uploaded figure has a “rev” extension reflecting this). As well, we annotated an additional important, statistically significant data point pair in this panel; collectively, the various annotated point pairs in Fig 6B make a strong case for the in vivo interactions of CPI-613 and TZ mimicking the in vitro results. We substantially revised our discussion of Fig 6 in the main text (lines 497-514) to further address the various concerns of reviewer 2 about the interpretation these data.

In response to reviewer 2’s request, we have increased the resolution of the figures containing the Fig 2C,D panels and the related supplementary figure panels (S2C,F Fig). This eliminates pixilation of these images at magnifications sufficient for visualization of all the captured features of the fluorescent images. Each of these two figures has an HD extension reflecting this.

Reviewer 2 asks about the definition of the term “homeostatic consumption.” We provide a definition of this term the first time it is used in the main text (lines 97-101). The gist is that CPI-613-induced accelerated flux through glycolysis or fatty acid beta-oxidation provides resources that can overcome/prevent the ATP depletion that would otherwise result from drug targeting of the TCA cycle as a result of by-passing this target.

Finally, Reviewer 2 has suggested various local changes to the text. We have accepted all these suggestions with minor exceptions, as follows (line numbers in original manuscript draft are shown in parentheses).

First, we have made the requested revisions to each of the following lines: 61-63 (57-59), 82-83 (77-81), and 88-90 (83-84), 107 (97), 229 (219), 281 (265), and Fig 5 legend 442-444 (410).

Second, lines in the original text, 154-157 and 313-323, were moved from the RESULTS to the DISCUSSION and revised [see lines 579-596 in revised text], as requested.

Third, however, we have left the segments represented by lines 184-188 (174-178), 192-195 (182-85) and 270-276 (254-260) in their original locations, rather than moving them to the DISCUSSION. We understand why the reviewer might view the content of these brief segments as appropriate for the DISCUSSION; nonetheless, we believe they serve an important function in their current locations to allow the reader to more readily follow the logic of the arguments and experimental design developed in the flow of the RESULTS section.

**REVIEWER 3:**

In response to an initial comment on the generalizability of lipid and glycogen stores determining PDAC CPI-613 sensitivity by reviewer 3, we have taken advantage of the exceptional sensitivity of the PC3 prostate cancer cell line to test this, as also noted above. We find the predicted very low levels of glycogen and lipid stores in this very sensitive line (see new figures S2E,F Fig; text lines 251-256). Collectively, our results robustly support the generality of the correlation between the levels of endogenous nutrient stores and CPI-613 sensitivity in carcinoma cells.

Reviewer 3 asks about the potential of lactate to rescue from CPI-613-induced cell death, in view of glucose rescue. In contrast to glutamine and glucose, lactate does not rescue from robust CPI-613 doses (80µM; see new S4D Fig). This outcome is expected in view of the properties of the shuttle delivering the extra lactate electron pair (relative to pyruvate) to the ETC, as explained and referenced in the added text (lines 402-407). Specifically, the lactate-malate-aspartate shuttle responsible for transfer of the extra lactate electrons to the ETC requires ongoing matrix metabolism of the pyruvate generated by the “reverse” LDH reaction to drive this shuttle in the forward direction (see reference 30 in the MANUSCRIPT). This matrix pyruvate metabolism is, of course, initiated by PDH and, thus, inhibited by CPI-613 (see reference 11 in MANUSCRIPT). As expected on this view, at lower CPI-613 doses lactate can produce modest rescue (S4D Fig), presumably reflecting a small amount of flux through PDH.

Reviewer 3 asks that we further clarify our current understanding of the levels of free glucose in the solid tumor environment, both in preclinical and clinical contexts. We have done this in lines 695-704.

Review 3 asks that we clarify the interpretation of several features of the in vivo TGI data in Fig 6, some initially unexpected. He/she also asks that we clarify the replication of the control data in the individual excerpts from a large TGI study (Fig 6B). We substantially revised the discussion of these results in response to these comments (lines 497-514). We have also revised the legend to Fig 6 and replotted the data in the lower left panel of Fig 6B to improve readability. Further, we annotated an additional significant, relevant point pair difference in this same panel; these revisions further clarify and emphasize the value of the evidence in this figure in addressing the issues in question.

Reviewer 3 asks that we comment on the significance of recent reports of promising CPI-613 single agent responses in Burkitt lymphoma clinical trials, in light of our results. We have done this in lines 705-711.
